# Giant Cerebral Aneurysm: Still Formidable Challenges in Modern Neurosurgery

**DOI:** 10.31662/jmaj.2022-0109

**Published:** 2022-06-24

**Authors:** Teiji Tominaga

**Affiliations:** 1 Department of Neurosurgery, Tohoku University Graduate School of Medicine, Miyagi, Japan

**Keywords:** Cerebral Aneurysm, Cerebral Infarction, Embolism, Surgery

A giant aneurysm is generally defined as an intracranial aneurysm with a diameter of 25 mm or more ^(1)^. Giant aneurysms are relatively rare, comprising ~5% of all intracranial aneurysms, and mostly present between the fifth and the seventh decades ^(2)^. They are slightly more common in females and are located in regions of higher blood flow velocity, such as the cavernous and supraclinoid carotid, vertebrobasilar, and basilar apex ^(2)^. Approximately 5%-10% present in the pediatric population. The natural history of a giant aneurysm is unfavorable. The rupture risk of intracranial aneurysms generally increases depending on its size. The annual risk of rupture of giant aneurysms reaches up to 33.4%, which is 76 times higher than that of aneurysms that are 3-4 mm in the largest dimension according to a large, prospective cohort study of unruptured cerebral aneurysms in the Japanese population ^(3)^. Thus, surgical treatment should be indicated for giant cerebral aneurysms. The primary aim of surgical treatment for giant cerebral aneurysms is the permanent exclusion of the aneurysm from the circulation with preservation of the blood flow of the incorporated branches and the secondary aim is the relief of the mass effect on the adjacent brain parenchyma or neural structure. These aims can be achieved by various surgical techniques, including direct clipping of the aneurysm neck or trapping of the aneurysm with or without bypass. Recently, endovascular treatment, such as coil embolization, stent-assisted coil embolization, or flowdiverter, has been applied to these challenging aneurysms, as the endovascular devices have become sophisticated. The current results of definitive surgical and endovascular treatments, even in the best centers, remain relatively poor compared with the improvements made in the results of treatment of smaller aneurysms. Most surgical series reported a surgical mortality of at least 6% and a morbidity of at least 20% ^(2)^. In the endovascular treatment, there is a significant risk of rebleeding after coil embolization and a relatively high incidence of recanalization ^(2)^. These difficulties are due to their physical size; incorporation of vessels and perforators; and often poor tissue characteristics, such as intra-aneurysmal thrombus and calcification of the aneurysmal wall.

Giant cerebral aneurysms sometimes accompany chronic intra-aneurysmal thrombus due to blood flow stagnation within the aneurysm. Serpentine aneurysms are a subgroup of thrombosed intracranial aneurysms defined as a partially thrombosed lesion with tortuous internal vascular channel. Giant serpentine aneurysms may show spontaneous complete thrombosis and subarachnoid hemorrhage ^(4)^. Tanaka reported a case with a giant middle cerebral artery aneurysm ^(5)^. The patient initially manifested cerebral infarction, which was followed by aneurysmal rupture. Computed tomography at presentation revealed high density within the aneurysm, which might suggest a thrombus formation within the aneurysm. The underlying mechanisms of this thrombus formation remain unclear. This might be due to secondary thrombus formation based on chronic existing serpentine-type thrombosis. This might also have resulted from an atherosclerotic occlusion of the distal branches of the middle cerebral artery. This speculation is based on the atherosclerotic changes in the contralateral middle cerebral artery. The third speculation is embolic occlusion of the aneurysm, although the embolic source is undetermined. This case is rare in terms of the second attack of aneurysmal rupture. Recanalization after the complete thrombosis of the aneurysm might affect the following fatal rupture, which could be induced by the use of antithrombotic therapy. Antithrombotic therapy is the primary standard treatment for ischemic stroke; however, its safety for patients with ischemic stroke harboring giant cerebral aneurysm remains unclear. This case might suggest that antithrombotic therapy for ischemic stroke secondary to giant cerebral aneurysm could lead to secondary rupture.

## Article Information

### Conflicts of Interest

None

### Disclaimer

Teiji Tominaga is one of the Editors of JMA Journal and on the journal’s Editorial Staff. He was not involved in the editorial evaluation or decision to accept this article for publication at all.
